# 

**Published:** 1899-05

**Authors:** 


### The Dental Society of the State of New York.

The Dental Society of the State of New York will hold its 31st annual meeting at Hotel Ten Eyck, Albany, May 10th and 11th. The session will begin promptly at 10 o'clock the first day, at which time it is desirable that every member be present, and especially a full representation of the delegates of the district societies.

The program is an exceedingly interesting one. Dr. Hales will present a paper extensively illustrated by microscopic projection, which will add very greatly to the general interest.

Headquarters will be at Hotel Ten Eyck, $3.50 per day.

There will be a large presentation of dental exhibits by the principal manufacturers of the country.

A cordial invitation is extended to all reputable members of the profession, of this and other States, to attend and participate in the discussion.

If any additional information is desired address Dr. Chas. S. Butler, of Buffalo, N. Y.

### NOTICES.

### International Dental Congress, Paris, 1900.

CHICAGO, April 25, 1899.

The undersigned committee appointed at Omaha by the National Dental Association at its meeting held August 30, 31 and September 1, 1898, will meet at Niagara Falls, New York, Tuesday, August 1, 1899, at 4 P. M. in the Cataract House to effect a permanent organization and transact any business which may come before it. You are expected to be present.

A. W. Harlan, Chairman, 1000 Masonic Temple, Chicago; T. W. Brophy, Chicago; A. H. Fuller, St. Louis; H. J. McKellops, St. Louis; J. Taft, Cincinnati; H. A. Smith, Cincinnati; W. W. Walker, New York; James McManus, Hartford; W. C. Barrett, Buffalo; B. Holly Smith, Baltimore; C. L. Goddard, San Francisco; L. L. Dunbar, San Francisco; W. E. Griswold, Denver; H. W. Morgan, Nashville; Frank Holland, Atlanta; E. C. Kirk, Philadelphia; J. D. Patterson, Kansas City; Thomas Fillebrown, Boston; Thomas E. Weeks, Minneapolis.

Please bring lists of those persons who may desire to read papers, give clinics, and be ready with suggestions which may facilitate the work of the committee in order to have a strong representation of the best element of the profession at the meeting. Any advance suggestions will be thankfully received from any one not able to be present at this meeting.

Yours truly, A. W. HARLAN.

### The Annual Meeting of the National Association Dental Faculties.

The annual meeting of the National Association Dental Faculties will be held Niagara Falls, beginning Friday July 28th, at 10 A. M. 1899, and continue 28th, 29th, 31st.

It is to be hoped there will be a full attendance, indeed that every dental college and member of the association will be represented, as much business of importance is to be transacted.

This is true every year, but this year it seems that more importance attaches to this meeting than any one hitherto.

The executive committee of this body will meet on Thursday, the 27th, at 10 o'clock for the preparation of business for the general meeting.

Any members having business with this committee will please be present at that time.

### EDITORIAL.

### THE DENTAL REGISTER,

**A MONTHLY MAGAZINE DEVOTED TO THE INTERESTS OF THE**

**DENTAL PROFESSION.**

*Terms Reduced to* $2.00 *Per Year. Single Copies,* 25 *Cents.*

**EDITED BY PROF. J. TAFT, D.D.S.**

—AND—

**Published by SAM'L A. CROCKER & CO., Ohio Dental and Surgical Depot.**

The volume commences in January and closes in December.

The REGISTER being issued monthly is always fresh, therefore Indispensable to the practitioner who desires to keep posted up with the new inventions and improvements which are daily developed. Neither expense nor effort will be spared to give value and variety to its contents Articles will be illustrated with well executed engravings whenever they will add to the interest or assist to explanation.

Its extensive circulation and frequency of issue make it one of the BEST DENTAL ADVERTISING MEDIUMS in the United States.

All subscriptions must begin with January or July.

Local or special notices, five lines or less, will be inserted for $3 each insertion; over five lines, 50 cts. per line.

All advertising bills payable in advance, or at furthest, after the issue of the first number containing the advertisement. All transient advertisements to be paid for in advance.

☞CONTRIBUTIONS SOLICITED.

Exclusively Editorial Communications should be addressed to the Editor.

The latest number of the REGISTER may be ordered with or without other good [illegible] any time, and all letters should be addres[illegible]

